# Frequency Mixing Magnetic Detection Setup Employing Permanent Ring Magnets as a Static Offset Field Source

**DOI:** 10.3390/s22228776

**Published:** 2022-11-14

**Authors:** Ali Mohammad Pourshahidi, Stefan Achtsnicht, Andreas Offenhäusser, Hans-Joachim Krause

**Affiliations:** 1 Institute of Biological Information Processing, Bioelectronics (IBI-3), Forschungszentrum Jülich, 52425 Jülich, Germany; 2 Faculty of Mathematics, Computer Science and Natural Sciences, RWTH Aachen University, 52062 Aachen, Germany; 3 Institute of Nano-and Biotechnologies (INB), FH Aachen University of Applied Sciences, 52428 Jülich, Germany

**Keywords:** magnetic nanoparticles, frequency mixing magnetic detection, biosensors, magnetic sensors

## Abstract

Frequency mixing magnetic detection (FMMD) has been explored for its applications in fields of magnetic biosensing, multiplex detection of magnetic nanoparticles (MNP) and the determination of core size distribution of MNP samples. Such applications rely on the application of a static offset magnetic field, which is generated traditionally with an electromagnet. Such a setup requires a current source, as well as passive or active cooling strategies, which directly sets a limitation based on the portability aspect that is desired for point of care (POC) monitoring applications. In this work, a measurement head is introduced that involves the utilization of two ring-shaped permanent magnets to generate a static offset magnetic field. A steel cylinder in the ring bores homogenizes the field. By variation of the distance between the ring magnets and of the thickness of the steel cylinder, the magnitude of the magnetic field at the sample position can be adjusted. Furthermore, the measurement setup is compared to the electromagnet offset module based on measured signals and temperature behavior.

## 1. Introduction

The use of magnetic nanoparticles (MNPs) and magnetic beads (MBs) in scientific research and industry has progressed throughout the years [1,2,3]. Extensive research is being done on synthesizing novel MNPs [4,5] and their applications; for example, hyperthermia [6,7,8,9,10], magnetic particle imaging (MPI) [11,12,13], drug delivery [14,15,16], separation [17] and finally, biosensing, where they are used as markers [18,19,20,21]. For all of these applications, the magnetic properties of the MNPs are of utmost importance. Hence, various techniques are employed to characterize the magnetic properties of the synthesized MNPs [22,23].

Susceptometric techniques have gained a lot of attention over the years for their role in biosensing applications as they show the capability to be used as a characterization tool, for example, in assessing the magnetic response of MNPs [24,25] and determining the hydrodynamic and core size distribution of the MNPs [26]. In many of these applications, it is necessary to employ a static offset magnetic field.

Frequency mixing magnetic detection (FMMD) technology has been exploited over the past fifteen years [27]. It presents an advantage due to its specificity to the nonlinear magnetic response feature of the superparamagnetic nanoparticles. Hence, it has been widely utilized in biosensor research for the detection of different biological entities; for example, to detect antibiotics in milk [28] and of aflatoxin B1 [29], which can be used as a quality control measure in agricultural and dairy farms. Moreover, the FMMD technique was also successfully utilized for the detection of the cholera toxin subunit B [30], C-reactive protein [31], Francisella tularensis [32] and other targets [18,33,34]. In [35], the offset-dependent FMMD signal has been shown to be utilizable for the binary mixture detection of magnetic bead types. Furthermore, it was also shown in [24] that it is feasible to utilize the offset-dependent FMMD signal for the characterization of magnetic nanoparticles with respect to their core size distribution.

The offset-dependent FMMD signal is typically measured using a setup comprising an electromagnet that generates the static offset magnetic field [35,36]. The FMMD with electromagnet offset module (EMOM) is a tabletop lab-based device since an external power supply is required to drive the electromagnet, and due to heat generation, an active cooling system is used to reduce the heat generated by the system. The EMOM setup allows the measurement of the offset-dependent FMMD signals through the computer control of the applied static offset magnetic field using a programmable current source. On the other hand, it has its own limitations, which are a lack of portability and cooling requirements.

In this paper, we report on a new measurement head design incorporating two ring-shaped permanent magnets for generating different static offset magnetic fields, which are needed to obtain offset-dependent FMMD signals. Furthermore, we compare the measurements performed with our permanent magnet with the results obtained with the electromagnet module. We assess the advantage of using the offset-dependent signals for the detection of magnetic nanoparticle samples with low concentration and show that by using a static offset magnetic field, the measurement sensitivity can be increased.

## 2. Materials and Methods

### 2.1. Frequency Mixing Magnetic Detection (FMMD)

FMMD is a technique that relies on the intermodulation of two separate alternating magnetic fields with low (*f*_2_)- and high (*f*_1_)-frequency spectral components. The time-dependent excitation magnetic field *B(t)* has a form of

(1) $$B\left(t\right)=B_{0}+B_{1}sin\left(2\pi f_{1}t\right)+B_{2}sin\left(2\pi f_{2}t\right)$$

where *B*_0_ is the magnitude of the static offset magnetic field, and *B*_1_ and *B*_2_ are the amplitudes of the applied high and low-frequency alternating magnetic fields, respectively. The ensemble of superparamagnetic nanoparticles is measured using the ensuing mixing harmonics to do selective quantification. In this method, the driving and excitation fields are two alternating magnetic fields that are applied to the superparamagnetic nanoparticles. The magnetization of the superparamagnetic nanoparticles approaches a saturation level when the driving field, with a frequency (*f*_2_) in the range of a few tens of Hertz, is strong enough, typically within the range of a few mT. On the other hand, the magnetization state of the superparamagnetic nanoparticles is further probed using the excitation field with the frequency (*f*_1_). The resulting magnetization response of the particle ensembles at the even and odd mixing harmonics at frequencies *f_1_+ n·f_2_*, (*n* ϵ *N*) is analyzed. In the absence of a static magnetic offset field (*B*_0_), one can only detect the odd mixing harmonics (*n* = 2, 4, …) since the Langevin function is point-symmetric, as described in detail in [27]. However, in the presence of a non-vanishing offset field, the even mixing harmonics (*n* = 1, 3, …) will also appear.

### 2.2. Experimental Setup

The experimental setup consists of an FMMD readout electronics, referred to as a magnetic reader, the electromagnet offset module (EMOM) and our newly developed measurement head employing two permanent ring magnets as the source of the static offset magnetic field, named permanent magnet offset module (PMOM).

#### 2.2.1. Magnetic Reader

We use the same in-house FMMD readout electronics as has been applied in previous studies [35,37]. It utilizes two AD9834 direct digital synthesis (DDS) chips from Analog Devices for the generation of the low-frequency and high-frequency excitation fields. The generated signals are then amplified through a set of power amplifiers of the Texas Instruments BUF634 type with an upper current limit of 250 mA to generate the high and low-frequency excitation magnetic fields. The response of the detection coil signal is initially low-pass-filtered and further amplified using an Analog Devices AD829 low-noise amplifier. The amplified signal is then digitized using the National Instruments NI-USB 6251 data acquisition card and further processed using a PC.

#### 2.2.2. Electromagnet Offset Module (EMOM)

The static offset FMMD utilizing an electromagnet as the source of an offset field was used to serve as a reference to compare the measurement results. Details of this measurement system have been reported in [35]. Additionally, to reduce measurement signal drifts in the electromagnet setup due to high temperatures, the temperature of the offset coil was reduced through a power management routine by applying the offset field in a pulsed fashion (EMOM-pulsed).

#### 2.2.3. FEM Simulations of PMOM Configuration

In the proposed configuration of PMOM to provide the static offset magnetic field, two axially magnetized ring-shaped ferrite permanent magnets are employed in such an orientation that they attract each other. To attenuate and homogenize the resulting magnetic field at the position of the sample, a hollow steel cylinder is utilized. Finite element method (FEM) calculations using the open-source software FEMM (version 4.2) were performed to initially study the behavior of the resulting magnetic field as a function of the distance between the two magnets and also the thickness of the steel cylinder. Ring magnets with an inner radius of 30 mm, an outer radius of 50 mm and a height of 20 mm were studied. The magnetic material with C5 quality was used for the simulations, which closely corresponds to the Y35 quality, according to the manufacturer [38]. The steel elements were simulated using the structural steel ST37. As the arrangement of the ring magnets and the steel cylinder were treated as a rotationally symmetric problem, a 2D simulation with an axisymmetrical problem in polar coordinates has been used.

#### 2.2.4. Measurement Head Design with Permanent Magnet

**Figure 1:**
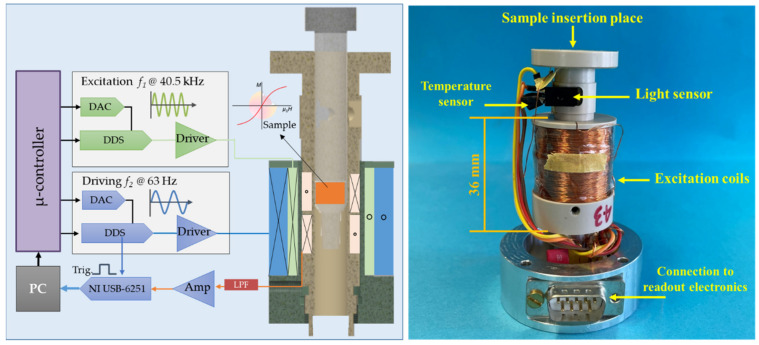
(**Left**) Schematic block diagram of the excitation and readout circuitry connected to the cross-sectional view of the measurement head assembly. (**Right**) Photo of the internal configuration of the measurement head and the high- and low-frequency excitation coils around the detection coils. The sample is inserted into the measurement head from above, and a light sensor is used to monitor the insertion and removal of the sample.

The measurement compartment of the FMMD technology comprises two coaxial coils utilized for the generation of the low and high-frequency excitation magnetic fields and a differentially wound detection coil that serves as a sensing compartment [27]. Figure 1 shows the excitation coils placed and adjusted around the detection coil compartment. A temperature sensor of type DS18B20 from Maxim Integrated is utilized to measure the internal temperature of the measurement head, and a light barrier sensor is used to react upon the sample insertion.

**Figure 2:**
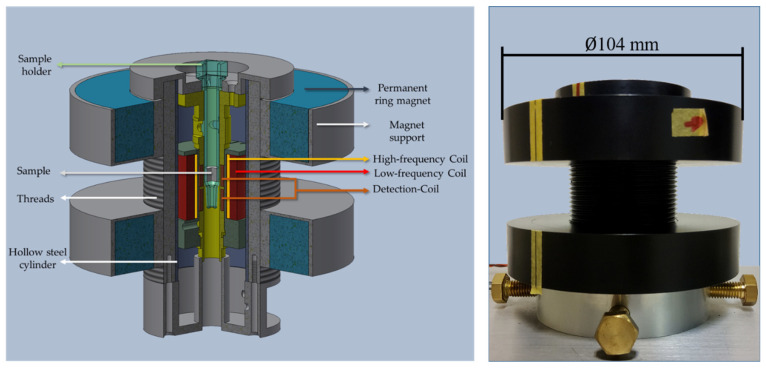
(**Left**) Schematic representation of the PMOM measurement head, the individual parts are labelled. (**Right**) The picture of the PMOM measurement head, which is held inside a place holder and fastened using three hexagonal screws.

The static offset magnetic field of this newly developed measurement head uses two ring-shaped ferrite permanent magnets with an internal diameter of 60 mm, an outer diameter of 100 mm and a thickness of 20 mm purchased from Webcraft GmbH (Gottmadingen, Germany). The schematic design of the measurement head is shown in Figure 2. Each of these ring magnets is placed in an aluminium ring-shaped support with a large inner thread. The inner thread of the supports is screwed onto the outer thread of the measurement head housing. This allows the distance between the two magnets to be adjusted through the rotation of the magnet supports. The supports are screwed in such a way that the bottoms face each other, preventing the magnets from coming into direct contact with each other. The attractive force between the two magnets holds the magnets inside their aluminium supports so that they don’t have to be glued or affixed otherwise.

By moving the magnets towards or away from each other, the static offset magnetic field at the sample position in the measurement head can be increased or decreased. Additionally, a hollow steel cylinder with an internal diameter of 35 mm and a wall thickness of 1.5 mm is inserted between the outer permanent magnets and the coil systems to serve as a magnetic field attenuator and homogenizer. The thickness of the steel cylinder also determines the magnitude of the static magnetic field at its inside. The thicker the steel, the weaker the magnetic field. Thus, the choice of thickness determines the minimum and maximum magnetic field, and in which range the static magnetic field can be adjusted.

### 2.3. Magnetic Nanoparticles

The superparamagnetic nanoparticles used throughout the experiments have been procured from micromod GmbH (Rostock, Germany). They are of the Synomag D type, with a hydrodynamic diameter of 70 nm with a plain surface and a particle concentration in the stock solution of 25 mg/mL.

## 3. Results and Discussion

In this section, we initially present the results of the FEM simulation showing the behavior of the magnitude of the magnetic field through the variation of both the distance between the ring magnets and the thickness of the steel cylinder. Following that, we show the characterization of the experimental setup, which was done through a set of experiments, also serving as further assessment for checking the practicality of the design. We present the characterization of the permanent ring magnets at different distances. Three modes of measurement (continuous-EMOM, pulsed-EMOM and PMOM) are then compared with respect to the FMMD signal change and temperature development. Additionally, we analyze the offset-dependent signal measured by using PMOM for a sample of MNPs and compare it with the measurement using the pulsed-EMOM setup. Finally, we assess if the offset-dependent signal at particular fields exhibits an advantage when utilizing it for the measurement of lower concentration MNPs.

### 3.1. Simulation Results

**Figure 3:**
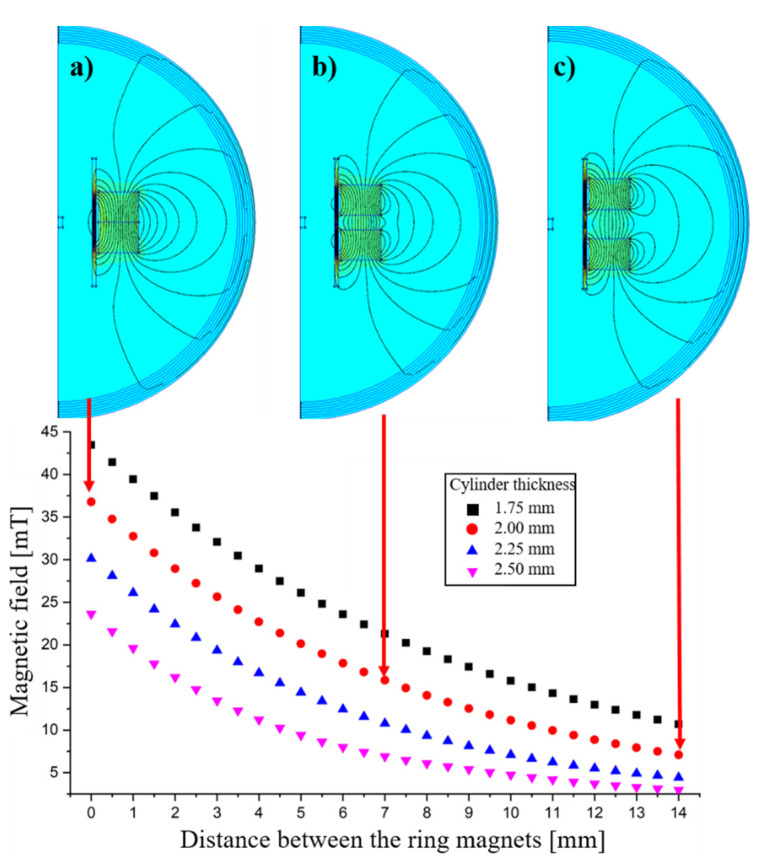
Simulated magnetic field as a function of the distance between the two magnets with steel cylinders of different thicknesses (color-coded). The magnets are moved symmetrically away from each other, as shown in (**a**–**c**).

Simulations, according to Section 2.2.3, were performed for the variation of the distance between the ring magnets from 0 to 14 mm for four different thicknesses of the steel cylinder (1.75, 2.00, 2.25 and 2.50 mm). The values at the center point of the rotational symmetry axis (0, 0) and the respective topographical images of the simulation were saved. Figure 3 depicts the variation of the magnetic field as a function of the distance between two ring magnets at the center point of the rotational symmetry axis, which is the location where the sample will be placed. The simulation images, shown as (a) to (c), highlight three arrangements (the complete effect of the variation is presented as animation in the Supplementary section): (a) when the two ring magnets are in direct contact with each other, (b) when the mid-region where the distance between the two ring magnets is half of the total distance (i.e., 7 mm) and (c) the farthest point of the simulation with a 14 mm distance between the two ring magnets. The complete effect of the variation is presented as an animation in the Supplementary section. The images belong to the simulations using the 2.00 mm cylinder thickness. One can see that when the magnets are most close to each other, corresponding to simulation picture (a), the largest magnetic field is obtained (in this case ~37 mT), and as the magnets move away from each other, the total magnetic field of the assessment point reduces until the final point of the simulation (inter magnet distance = 14 mm), where we obtain ~7.5 mT. Another observation that one could obtain from the presented results is that, as expected, the magnetic field attenuates upon increasing the thickness of the steel cylinder (color-coded in Figure 3). The results of the simulations show that it is possible to select the accessible magnetic field range at the sample location by choosing the thickness of the steel cylinder. The exact field value can then be adjusted by variation in the distance between the ring magnets. The steel cylinder also improves the homogeneity of the field at the sample position.

### 3.2. Measurement Head Excitation and Static Offset Field Characterization

**Figure 4:**
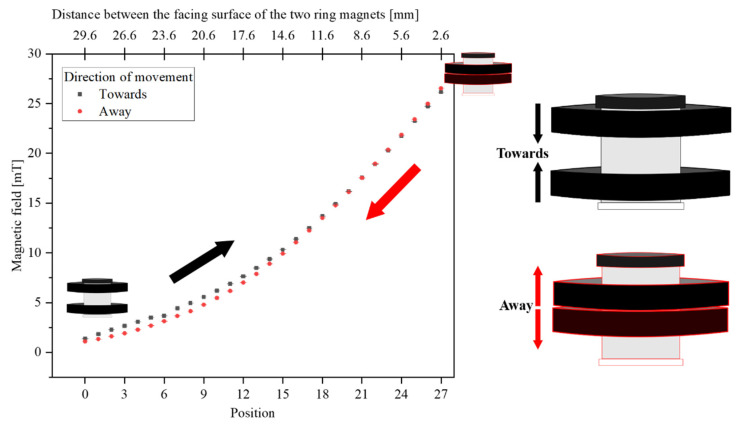
The dynamic applicable static magnetic field through PMOM. The magnetic fields were measured in 28 steps by moving the two permanent ring magnets towards each other (black squares following the black arrow), thus increasing the magnetic field, and then pulling them away from each other to decrease the field (red circles following red arrow).

The magnetic field generated by the ring magnets was measured using a Hall sensor (Allegro A1324) read out with a voltmeter. The Hall sensor was placed inside a custom 3D printed holder, which positions the sensor in the measurement head so that it was at the position of the real MB sample. The ring magnets were adjusted symmetrically through rotation around the threaded body of the housing (see Figure 2). Figure 4 shows the measured magnetic field in 28 steps with a step width of 1 mm, the black squares represent the measurements taken while the magnets are moving towards each other, and the red squares show the measured data when the magnets move away from each other. From this, we obtain the dynamic range of applicable static offset magnetic fields, starting from 2 mT (at level 0) when the magnets are 29.6 mm apart from each other, until 26.5 mT (at level 27) at the closest possible position of the two magnets, just 2.6 mm apart from each other (determined by twice the thickness of the aluminium support holding each magnet). Additionally, as can be seen in the graph, we observe hysteresis between levels 0 to 15 due to the presence of the steel cylinder, which has ferromagnetic properties and therefore exhibits hysteresis. This cylinder is needed as it is used for the attenuation and homogenization of the magnetic field. Thus, one has to keep in mind that for reaching a particular field value, the direction of the movement is important. Therefore, one should always adjust the measurement distance by approaching it from the same side. In our experiments, we measured the static magnetic field after each change of the magnet distances before inserting the sample.

The magnitudes of the sinusoidal excitation magnetic fields generated by the excitation coils inside the measurement head were also measured. The low-frequency excitation coil is set to yield a magnetic field amplitude of *B*_2_ = 16.5 mT with a frequency of 63 Hz, and the high-frequency excitation coil produces a magnetic field amplitude of *B*_1_ = 1.2 mT with a frequency of 40.5 kHz. These field amplitudes are comparable to the EMOM setup with identical coil dimensions where we measured *B*_1_ = 1.29 mT and *B*_2_ = 16.4 mT. The small deviations can be attributed to manufacturing tolerances.

### 3.3. Static Offset Dependant Measurement Signal

To evaluate the static offset-dependent FMMD signal, we measured a magnetic nanoparticle sample of type Synomag D 70 nm in an immobilized state. The immobilization was performed according to the method presented in [24]. The sample was initially measured using the PMOM setup, and then the same sample was measured using the FMMD-EMOM setup in pulsed and continuous mode.

The background signal and the signal generated due to the presence of the sample were measured at each offset level. After measurement and background subtraction, the phase correction of the signals was performed in the complex domain by the projection of the measured signals to the real axis.

**Figure 5:**
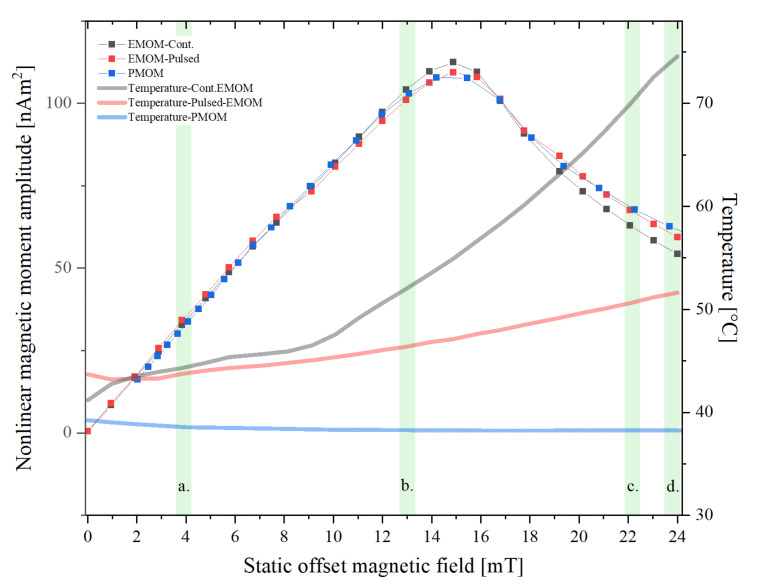
Nonlinear magnetic moment trace of sample Syn70 measured with the PMOM setup (blue squares), and with the EMOM setup in pulsed (red squares) and continuous (black squares) mode over a field range of 0 to 24 mT, for mixing harmonic *f*_1_ + *f*_2_. The temperature development in the measurement head is plotted for each case as faded solid lines with matching colors. The signal values of the different setups for four different regions (a–d, marked in green) are given in Table 1.

The temperature development comparison and its impact on the measurement signal were assessed through a comparison of the measurement signal of the first even frequency mixing harmonic (*f*_1_ *+ f*_2_). In the case of the EMOM setup, the offset range was varied from 0 to 24 mT in steps of 1 mT, and in the case of PMOM, the full dynamic range from 2 mT to 26.5 mT, as mentioned above, was used. For comparison in Figure 5, the static offset magnetic field range is set to cover 0 to 24 mT. The measurements done with EMOM-continuous, EMOM-pulsed and PMOM are presented as solid squares in black, red and blue, respectively, and are connected as a visualization aid. The respective temperature development in the measurement head has been plotted in the same graph using solid lines with matching colors. We can see that the temperature reaches almost 75 °C at the maximum field at the end of the scan in the case of continuous mode EMOM. However, using the pulsed mode, the temperature variation was substantially reduced by ~23 °C. On the other hand, there was practically no change in the temperature observed in the case of permanent magnets, as expected. The temperature fluctuated just weakly around the initial temperature of the system. This shows that the temperature increase in other modes is not due to the FMMD part of the measurement head but rather because of the contribution of the electromagnet. For comparison, four different sections have been marked in the graph, labelled a. to d., to observe the trend of signal change due to the temperature drift of the system. The values are listed in Table 1.

The temperature effect on the FMMD measurement results is out of the scope of this article; however, the issue needs to be briefly addressed. We compared the pulsed and continuous modes of EMOM since the static offset magnetic field was the same in both cases. We observed that in the region where the temperatures of both modes starts to deviate but are still very close to each other, the absolute variation in the measured amplitude was ~1.3 nAm^2^. The variations in regions b, c and d reached 3, 4.5 and 5 nAm^2^, respectively. On the other hand, the temperature variations were minimal in the PMOM setup. Comparing the EMOM-pulsed and PMOM in the same regions, we noted a maximum absolute deviation of less than 3 nAm^2^. It needs to be taken into account that the setups do not yield the exact same magnetic fields and that the highest deviations are observed at the points where the static magnetic offset field is slightly different.

**Figure 6:**
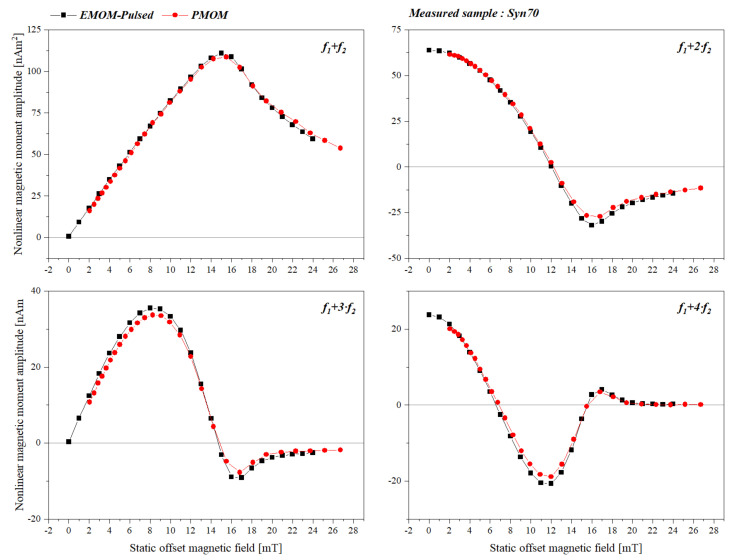
Nonlinear magnetic moment trace of sample Syn70 measured with EMOM−pulsed setup (black squares) over a field range of 0 to 24 mT and the PMOM setup (red circles) over a field range of 2 to 26.5 mT, for mixing harmonics *f*_1_ *+ f*_2_*, f*_1_ *+* 2*·f*_2_*, f*_1_ *+* 3*·f*_2_ and *f*_1_ *+* 4*·f*_2_.

For further comparison, we used the measured data. Figure 6 shows the traces of the first four frequency mixing harmonics (*f*_1_ *+ f*_2_*, f*_1_ *+* 2*·f*_2_*, f*_1_ + 3*·f*_2_ and *f*_1_ *+* 4*·f*_2_) for the measurement sequences of the pulsed-EMOM setup and the PMOM setup. The solid squares indicate the measurements performed using the pulsed-EMOM setup, and the solid red circles show the measurements performed using the PMOM setup.

For comparison, the corresponding field data of PMOM was extracted by doing a spline fit and calculating the total mean percentage error to the EMOM-Pulsed data. One can see that both measurements are in good agreement which each other, with a mean percentage error of 4.5%.

A good way to compare the measurement signals obtained from these two systems with each other is to compare the location of the features of the FMMD signals (i.e., the extremums and zero-crossings). The locations of the extremums of the measured sample with two systems are presented in Table 2. If we consider the electromagnet setup as a reference, we can see that the feature locations measured by the permanent magnet show a deviation of less than 1.5%. The observed deviations are in the acceptable range, however, the reason behind these small deviations could be due to small differences in the excitation field amplitudes of the two systems, and the different system temperatures at which the sample was measured.

### 3.4. Sensitivity Comparison

As mentioned earlier, one of the areas where FMMD is extensively used is the field of biosensors. In bio-sensing applications, it is often important to measure very low concentrations of the analyte (e.g., in sandwich immunoassays). It is common in FMMD to use the amplitude of frequency mixing component *f*_1_ *+ 2·f*_2_ to measure and determine the concentration of the analyte as it is the largest non-vanishing component without a static magnetic field. However, as can be seen from Figure 6, top left and right, the mixing component *f*_1_ *+ f*_2_ in its maximum at ~15 mT offset field is approximately 1.6 times larger than the *f*_1_ *+* 2*·f*_2_ component at 0 mT. Therefore, to be able to lower the detection limit in order to attain a better sensitivity, it would be advantageous to measure the *f*_1_ *+ f*_2_ component at *B*_0_ *=* 15 mT instead of measuring the usual *f*_1_ *+* 2*·f*_2_ component without an offset field.

## 4. Conclusions

Offset-dependent FMMD signals are utilized in many applications. Due to power requirements, most FMMD modules with offset fields are lab-based due to the incorporation of an electromagnet as the offset source and the required cooling strategies. A new measurement head was designed and constructed, incorporating ring-shaped permanent magnets for the measurement of static offset field-dependent FMMD signals. The behavior of the resulting magnetic field of such an arrangement was initially studied through FEM simulations, thus confirming the expectations and serving as a guide for building the experimental setup. After characterizing this measurement head, it was compared with a standard FMMD setup incorporating an electromagnet for generating a static offset magnetic field, using active cooling and a power management cooling strategy. As expected, the analysis yielded that the PMOM setup shows an almost constant temperature during the measurement process, independent of the magnetic offset field. Furthermore, a comparison was performed between the pulsed-EMOM and PMOM by measuring an immobilized magnetic nanoparticle sample of Synomag D 70 nm. The variation among signals was determined to be 4.5% which is in an acceptable range. Moreover, utilizing the measurement signal *f*_1_ *+ f*_2_ at a specific magnetic offset field, where the maxima are occurring, yields a 60% higher signal, and therefore provides an advantage in measuring lower concentrations than using the typical *f*_1_ *+* 2*·f*_2_ harmonics without an offset field. This feature could be applied for future FMMD sensitivity enhancement. However, one has to consider the limitations of the current module as well. The PMOM is bulkier than the standard handheld device. Further developments are required to automatize the measurement procedure and to enhance the user-friendliness and portability of the device for in-field applications.

## Figures and Tables

**Table 1:** Temperatures and nonlinear magnetic moment amplitudes of the three measurement modes at different regions.

Region	Temperature [°C]			Nonlinear Magnetic Moment Amplitude [nAm^2^]		
	EMOM-Pulsed	EMOM-Cont.	PMOM	EMOM-Pulsed	EMOM-Cont.	PMOM
**a**	43.75	44.31	39.00	34.16	32.84	33.73
**b**	46.37	52.10	38.60	101.03	104.07	103.03
**c**	50.62	69.90	38.25	67.56	62.97	67.83
**d**	51.60	74.56	38.50	59.41	54.33	62.714

**Table 2:** Comparison between the locations of the characteristic features of the sample Syn70 measured with EMOM and PMOM setup.

Mixing term	Feature	Syn70-EMOM Feature Location [mT]	Syn70-PMOM Feature Location [mT]	Difference [%]
**f_1_ + f_2_**	Maximum	15.06	14.95	0.73
**f_1_ + 2·f_2_**	Zero	12.03	12.2	1.41
	Minimum	16.13	16.36	1.42
**f_1_ + 3·f_2_**	Maximum	8.35	8.41	0.71
	Zero	14.60	14.83	1.5
	Minimum	16.56	16.80	1.4
**f_1_ + 4·f_2_**	1st Zero	6.57	6.65	1.2
	Minimum	11.54	11.52	0.17
	2nd Zero	15.57	15.63	0.38
	Maximum	16.99	17.11	0.70

## Data Availability

The data presented in this study are available on request from the corresponding author.

## Supplementary Materials

The following supporting information can be downloaded at: https://www.mdpi.com/article/10.3390/s22228776/s1. Video S1: FEM Simulation of varying static offset magnetic field.gif.
