# Supplementary figures and images for: Frequency Mixing Magnetic Detection Setup Employing Permanent Ring Magnets as a Static Offset Field Source
Source: Sensors (Basel). 2022 Nov 14;22(22):8776. doi: 10.3390/s22228776 (PMC9694433; doi:10.3390/s22228776)

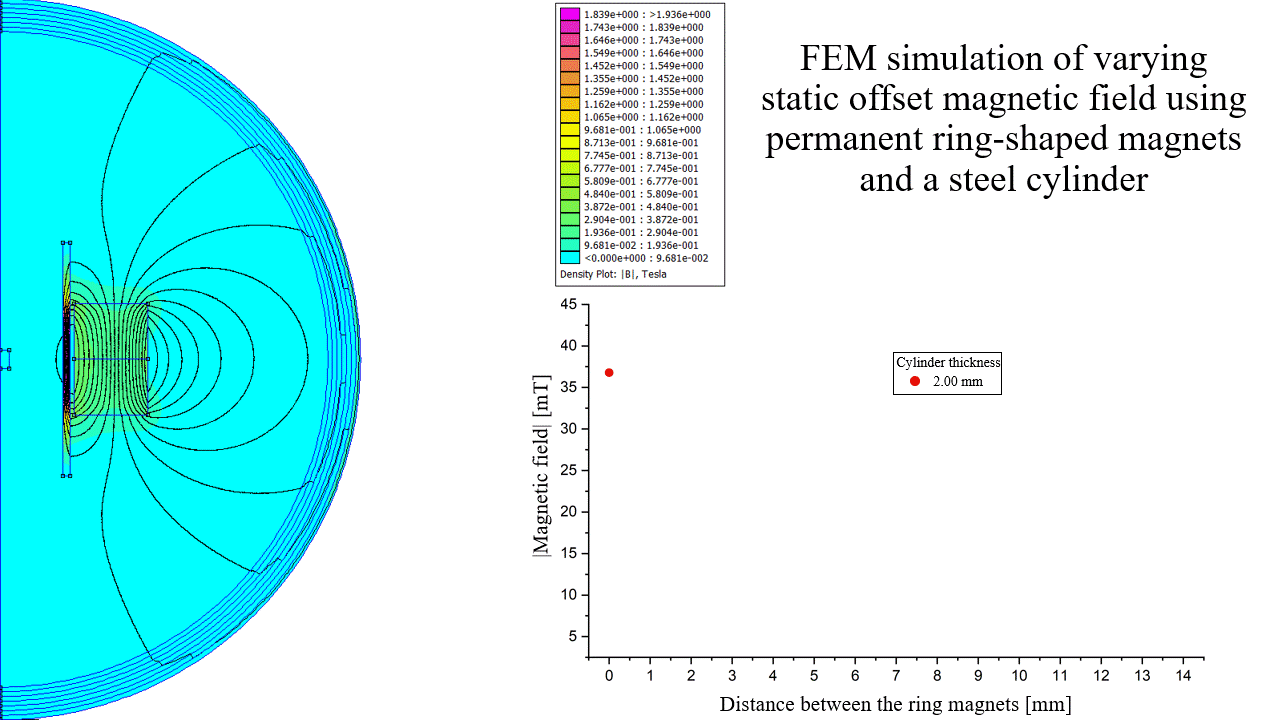

Supplement: Supplementary file 1 [file sensors-22-08776-s001.zip › sensors-2009520-supplementary-11.14.gif]
